# Thermal Stability of Glucokinases in *Thermoanaerobacter tengcongensis*

**DOI:** 10.1155/2013/646539

**Published:** 2013-08-24

**Authors:** Zhong Qian, Jingjing Zhao, Xue Bai, Wei Tong, Zhen Chen, Hanfu Wei, Quanhui Wang, Siqi Liu

**Affiliations:** ^1^Beijing Institute of Genomics, Chinese Academy of Sciences, No. 1 Beichen West Road, Chaoyang District, Beijing 100101, China; ^2^Developmental Genetics Section, Laboratory of Molecular Biology, Center for Cancer Research, National Cancer Institute, National Institutes of Health, Bethesda, MD 20892-4264, USA; ^3^Beijing Proteomics Innovation, Beijing Airport Industrial Zone B-6, Shunyi, Beijing 101318, China

## Abstract

In the genome of *Thermoanaerobacter tengcongensis*, three genes belonging to ROK (Repressor, ORF, and Kinase) family are annotated as glucokinases (GLKs). Using enzyme assays, the three GLKs were identified as ATP-dependent GLK (ATP-GLK), ADP-dependent GLK (ADP-GLK), and N-acetyl-glucosamine/mannosamine kinase (glu/man-NacK). The kinetic properties of the three GLKs such as *K*_*m*_, *V*_max_, optimal pH, and temperature were characterized, demonstrating that these enzymes performed the specific functions against varied substrates and under different temperatures. The abundance of ATP-GLK was attenuated when culture temperature was elevated and was almost undetectable at 80°C, whereas the ADP-GLK abundance was insensitive to temperature changes. Using degradation assays, ATP-GLK was found to have significantly faster degradation than ADP-GLK at 80°C. Co-immunoprecipitation results revealed that heat shock protein 60 (HSP60) could interact with ATP-GLK and ADP-GLK at 60 and 75°C, whereas at 80°C, the interaction was only effectively with ADP-GLK but not ATP-GLK. The functions of GLKs in *T. tengcongensis* are temperature dependent, likely regulated through interactions with HSP60.

## 1. Introduction

*Thermoanaerobacter tengcongensis* (*T. tengcongensis*) is a kind of thermophilic eubacteria, which is capable of survival from 50 to 80°C, with the optimal growth temperature of 75°C [1]. The survival mechanism of such kind of thermophilic bacteria has drawn great attention, due to their potential utilization in industry and elucidation of life evolution. The genome sequencing of *T. tengcongensis* has been finished in as early as 2002 [2], and its proteome has been surveyed in multiple views [3–6], which makes *T. tengcongensis* an ideal model for thermophilic mechanism study. 

Sugar kinases, such as glucose kinases (GLKs) are very important proteins involved in catalyzing the phosphorylation of glucose to glucose-6-phosphate, the key step of glycolysis, by which ATP is generated and help bacteria adapt to environmental changes. Many bacterial sugar kinases are grouped into ROK family, which is a large collection of sugar metabolism-related kinases, repressors, and some uncharacterized gene products [7]. According to the genome annotation towards *T*. *tengcongensis*, there are five genes predicted to be ROK members: *TTE0090*, *TTE0761*, *TTE1926*, *TTE1961*, and *TTE2418*. Of these genes, *TTE0090* was found to have the ability of phosphorylating glucose [8], and *TTE1926* was identified as the regulator of *gal* operon [9, 10]. Multisequence alignment analysis revealed that *TTE1961* and *TTE2418* share the conserved motifs of the glucokinases (GLKs) identified in *Thermotoga maritima *[11], whereas *TTE0761* does not have sequence similarity to any known GLK genes. *TTE0090*, *TTE1961* and *TTE2418* are thus presumably to be GLK or GLK-like genes. The questions arise consequently is why *T*. *tengcongensis* need three GLKs? Do they all perform the same function(s)? Do they follow a similar catalysis way to the sugar substrate(s)? These questions prompted us to initiate the study exploring the molecular mechanisms relevant to multiple enzymes with the similar function(s) in one bacterium, which will further help us understand the thermophilic mechanism of *T*. *tengcongensis*.

A number of GLKs have been identified, characterized, and crystallized both in prokaryotes [12–14] and eukaryotes [15–17]. Generally speaking, GLKs phosphorylate glucose utilizing ATP as the phosphoryl donor, termed ATP-dependent GLKs (ATP-GLKs). Besides that, microbial species contain the other two kinds of GLKs, ADP-dependent GLKs (ADP-GLKs) [18–20] and polyphosphate-dependent GLKs (poly(P)-GLKs) [21, 22], which utilize ADP and polyphosphate as the phosphoryl donors, respectively. Different types of GLK distribute in different species. Dörr et al. [23] investigated glucose phosphorylation in *Thermoproteus tenax* and found it had an ATP-dependent GLK with broad substrate specificity, while *Pyrococcus furiosus*, another kind of hyperthermophilic archaeon, was found having an ADP-dependent glucokinase [24]. Intriguingly, *Pyrococcus furiosus* has another kind of hexokinase, an ATP-dependent galactokinase, to which it was deduced reflecting the adaptation to a relatively low intracellular ATP concentration [25]. Generally, ADP is much more stable than ATP at elevated temperatures, with half lives of 750 min and 115 min at 90°C, but it is not clear why some hyperthermophilic species, such as *Thermotoga maritima* with optimal growth temperature about 80°C are still using ATP-dependent GLK. The adaptation mechanism for the ATP- and ADP-GLKs needs to be clarified.

In the current investigation, we first experimentally identified and distinguished the three candidates of GLKs in *T*. *tengcongensis* and further characterized their parameters of enzyme kinetics and optimal temperatures. Using Western blot analysis, the abundance of ATP-GLK exhibited a temperature-dependent attenuation, while ADP-GLK expression level remained constant. Compared to ATP-GLK, protein degradation of ADP-GLK was significantly lower at 80°C, both *in vitro* and *in vivo*. Co-immunoprecipitation assays further revealed that the instability of ATP-GLK at 80°C was likely to attribute to weak interactions of ATP-GLK with heat shock protein 60 (HSP60). Altogether, we come to a deduction that the function of ATP- and ADP-GLK in *T*. *tengcongensis* is partially regulated by the temperature-dependent interactions between GLKs and HSP60.

## 2. Materials and Methods

### 2.1. Bacteria Culture

*T. tengcongensis* strain MB^4T^ (T = type strain) was cultured in modified MB medium, as described previously [8]; all the cells were grown in anaerobic glass bottles. 1 mL of bacteria spore was activated by overnight incubation in 10 mL MB medium, and then 2 mL cells in log phase were added to every 100 mL medium for further culture. For temperature consistent culture, cells were inoculated into freshly prepared medium and cultured at the indicated temperatures to a stationary phase for collection. For the temperature-shift assay, cells were first cultured at 75°C to log phase and then quickly transferred to 80°C for further culture with indicated growth times. All the cell cultures were collected at 4°C with 5,000 rpm for 15 min. After washing with PBS twice, pellets were stored at −80°C for further use.

### 2.2. Enzyme Assay

The assay for standard enzyme activities was described previously [8]. The phosphorylation of glucose was detected by coupling the reaction to the reduction of NADP^+^ via 6-P-glucose dehydrogenase (G6PDH). Briefly, recombinant GLK protein (0.6 mM) was added to the reaction buffer (including 0.1 M Tris-HCl, pH 8.0, 15 mM ATP, 20 mM glucose and 6 mM Mg^2+^) which was preincubated at 75°C for 5 min. After 20 min of incubation, the reactions were terminated by cooling on ice. Then, the cooling mixture was added to the other reaction buffer (including 0.1 M Tris-HCl, pH 8.0, 10 mM NADP and 100 U/mL G6PDH). The increase in absorption at 340 nm (NADPH) was measured to get the enzyme activity by spectrophotometer.

The phosphorylation of other different sugars was measured by coupling the reaction to the oxidation of NADH via pyruvate (PK) and lactate dehydrogenase (LDH). Recombinant GLK protein (0.6 mM) was added to the reaction buffer (including 0.1 M Tris-HCl, pH 8.0, 15 mM ATP, 20 mM specific sugar, and 6 mM Mg^2+^) which was preincubated at 75°C for 5 min. After 20 min of incubation, the reactions were terminated by cooling on ice. Then, the cooling mixture mentioned above was added to the other reaction buffer (including 0.1 M Tris-HCl, pH 8.0, 1.5 mM KCl, 5 mM PEP and 0.5 mM NADH). At last, PK and LDH (700 and 1000 U/mL) were added to initiate the reaction.

For temperature-dependent enzyme activities analysis, enzymes were measured between 30 and 95°C using equal amount of sugars and coenzymes. For the analysis of the phosphoryl donors, the enzyme activities were measured in the standard assays at the optimal temperatures with equal concentration of different phosphoryl donors (ADP, ATP, CTP, GTP, ITP, and PEP). For the analysis of the metallic ion specificity, the enzyme activities were measured in the standard assays at the optimal temperatures with equal concentration of different metallic ions (Ca^2+^, Cu^2+^, Mg^2+^, Ni^2+^, Sr^2+^ and Zn^2+^). For the analysis of the sugar specificity, the enzyme activities were measured in the standard assays with equal concentration of different sugars (glucose, mannose, fructose, galactose, 2-deoxyglucose, gluNac, galNac, and manNac). For the analysis of thermal stability, the enzymes in sealed vials were incubated at the stated temperatures for 0 to 120 min. The vials were then cooled on ice for 10 min and detected the enzyme activity at the respective optimal temperatures [11]. All the data were processed by SigmaPlot software.

### 2.3. Co-Immunoprecipitation Analysis of Protein Interactions

Native protein lysates of *T*. *tengcongensis* were prepared as described previously [9]. Cells were cultured at the indicated temperatures to a stationary phase and then collected by centrifugation. The pellets were then washed and resuspended in 50 mM Tris-HCl (pH 7.2) containing 2 mg/mL of lysozyme. After incubation at 37°C for 1-2 h and centrifugation at 5000 rpm for 15 min, the supernatant was collected and preincubated with protein A beads. Sequentially, the supernatant was added to the slurry containing protein A beads and antibodies to ATP-GLK or ADP-GLK for an overnight incubation. After thorough washing with lysis buffer (50 mM Tris-HCl, pH 7.2), the immunoprecipitated complexes binding to the antibody were dissolved in SDS loading buffer (50 mM Tris-HCl, pH 6.8, 100 mM DTT, 2% SDS, 0.1% bromophenol blue, and 10% Glycerol) and detected by Western blot.

### 2.4. Protein Degradation Assay

A protein degradation assay was performed *in vitro* and *in vivo*. The *in vitro* analysis was done as described previously [9]. Recombinant proteins of ATP-GLK and ADP-GLK tagged with His were incubated with native protein lysates at the indicated temperatures for the stated times, respectively, followed by Western blot analysis with anti-His tag antibody. For the *in vivo* analysis, *T*. *tengcongensis*, which has been cultured at 75°C to log phase, was quickly transferred to 80°C for further culture for the stated times and finally collected to extract proteins for Western blot analysis using antibodies to *T*. *tengcongensis* ATP- and ADP-GLK, which were prepared by Beijing Protein Innovation.

### 2.5. *In Vitro* Protein Interaction Analysis

Purified recombinant ATP- and ADP-GLKs were incubated with recombinant HSP60, which was 6-His-tagged and highly expressed in *E*. *coli*, at the given temperatures for 30 min. The mixture was then cooled to room temperature and added to the HSP60 antibody-protein A complex for co-immunoprecipitation. After washing with lysis buffer, the precipitated products were eluted and examined by Western blot with anti-His tag antibody.

## 3. Results 

### 3.1. Kinetic Behaviors of the GLK Candidates *In Vitro*

*TTE0090 *was previously reported as GLK to catalyze glucose phosphorylation, while homology analysis of the amino acid sequences of *TTE1961 *and *TTE2418 *revealed the conserved GLK motifs in these two genes as shown in Supplementary Material available online at http://dx.doi.org/10.1155/2013/646539 (Supplementary Figures 1(A) and 1(B)). To understand the kinetic behaviors of all the three GLK candidates, their catalysis was evaluated with glucose as a shared sugar substrate and ATP or ADP as coenzyme. Both recombinant *TTE0090* and* TTE2418 *displayed significant transfer of phosphate from ATP to glucose, whereas recombinant *TTE1961 *did not have any similar activity. Furthermore, under the same reaction conditions but with ADP substituted for ATP, *TTE0090 *and *TTE2418* did not exhibit enzymatic activity, whereas *TTE1961* showed obvious catalysis of glucose phosphorylation. The primary data of enzymatic activity implies that all three candidates are capable of performing catalysis of glucose phosphorylation, utilizing glucose as substrate and selectively adopting the different phosphoryl donors, ATP, or ADP. 

The extensive investigations of kinetics of the three GLKs were carried out under different reaction conditions, and the results are summarized in Table 1. Regarding substrate specificity, although the three enzymes are able to catalyze glucose phosphorylation, their catalytic efficiencies are quite different. With ATP as the phosphoryl donor, *TTE0090 *has a higher catalytic efficiency to glucose (*K*_*m*_ = 2.4 mM), whereas *TTE2418 *has lower catalytic efficiency to glucose (*K*_*m*_ = 179.5 mM). With ADP as the phosphoryl donor,* TTE1961 *shows a comparable catalytic efficiency to that of *TTE0090* to glucose (*K*_*m*_ = 2.1 mM). Under physiological conditions, the concentration of glucose in a bacterium is almost impossible to accumulate to as high as 180 mM; thus, *TTE2418 *may not effectively utilize glucose as a substrate *in vivo*. Further experiments with alternative sugar substrates demonstrated that *TTE2418*, but not *TTE0090* or *TTE1961*, effectively catalyzed phosphorylation of N-acetylglucosamine (gluNac) at a lower *K*_*m*_ (0.9 mM), indicating that the native substrate for *TTE2418* was not glucose but possibly gluNac. Regarding the coenzyme specificity, *TTE0090* can adopt almost equal affinity to ATP (*K*_*m*_ = 2.6 mM) and GTP as the phosphoryl donor, while *TTE2418 *has a higher affinity to ATP (*K*_*m*_ = 1.9 mM) but a low affinity to GTP. *TTE1961*, on the other hand, has a negative binding to ATP but higher affinity to ADP (*K*_*m*_ = 0.9 mM). For all the three enzymes, the optimal cation was Mg^2+^, suggesting that the regulation mechanism of enzymatic activity through metal ions was similar in these enzymes. The substrate specificity for each GLK is summarized in Table 1. Of the 8 substrates tested, *TTE0090* favors glucose and mannose as substrates and poorly catalyzes the phosphorylation of 2-deoxyglucose; *TTE1961* effectively utilizes glucose and cannot efficiently catalyze mannose, and 2-deoxyglucose; while *TTE2418 *prefers to catalyze the phosphorylation of gluNac and N-Acetyl-D-mannosamine (manNac) and has relatively lower catalysis of glucose, mannose and galactose. Therefore, *TTE0090*, *TTE1961*, and* TTE2418 *were termed as ATP-GLK, ADP-GLK, and glu/man-NacK, respectively. 

**Figure 1:**
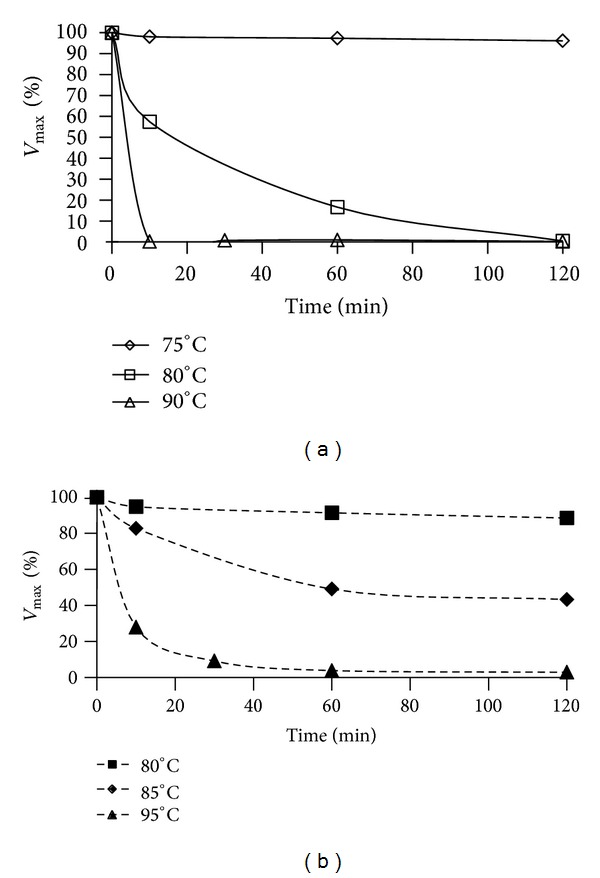
Thermal stability of recombinant ATP-GLK (a) and ADP-GLK (b). Recombinant ATP or ADP-GLK (0.6 mM) was incubated in 0.1 M Tris-HCl (pH 8.0) between 75 and 90°C (for ATP-GLK) or between 80 and 95°C (for ADP-GLK). One hundred percent of activity corresponded to the specific activity at 75°C (ATP-GLK) or 80°C (ADP-GLK) at the time of 0 min.

The temperature-dependent kinetics revealed that ATP-GLK and ADP-GLK held their catalytic activity with the optimal temperatures at 75 and 80°C, respectively (Supplementary Figure 2). Intriguingly, the two GLKs displayed very different thermal stabilities. As shown in Figure 1, the enzyme activity of ATP-GLK is gradually attenuated during incubation at 80°C and of ADP-GLK is relative stable under the same condition. Furthermore, ATP-GLK completely loses its activity after incubation of 10 min at 90°C, whereas ADP-GLK remains approximately 30% of activity after incubation 10 min at 95°C. Generally, enzyme activity is decided by the concentrations of substrate and protein and the configuration of active site. Under such enzyme assay conditions, the concentrations of glucose, ATP or ADP, and ATP-GLK or ADP-GLK remained consistent at all the temperatures. A logical deduction is that the protein configuration takes the corresponding changes due to temperature elevated, and the alternated structure may result in the GLK activity loss, especially for ATP-GLK.

### 3.2. The ATP- and ADP-GLK Abundances in *T*. *tengcongensis *Response to Temperature Changes

**Figure 2:**
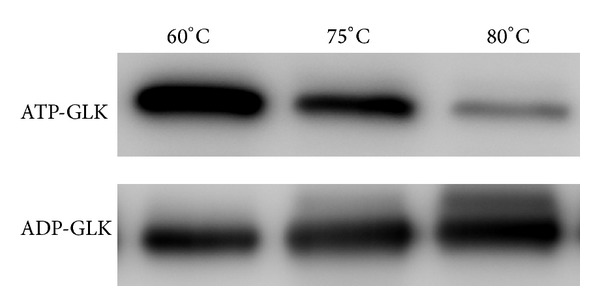
Western blot analysis of ATP- and ADP-GLK at different temperatures. *T*. *tengcongensis* cells cultured at different temperatures were collected and disrupted by ultrasonication. Equal amounts of *T*. *tengcongensis* soluble protein lysate (20 g) were loaded for SDS-PAGE separation followed by Western blot using antibodies against ATP- and ADP-GLK, respectively.

**Figure 3:**
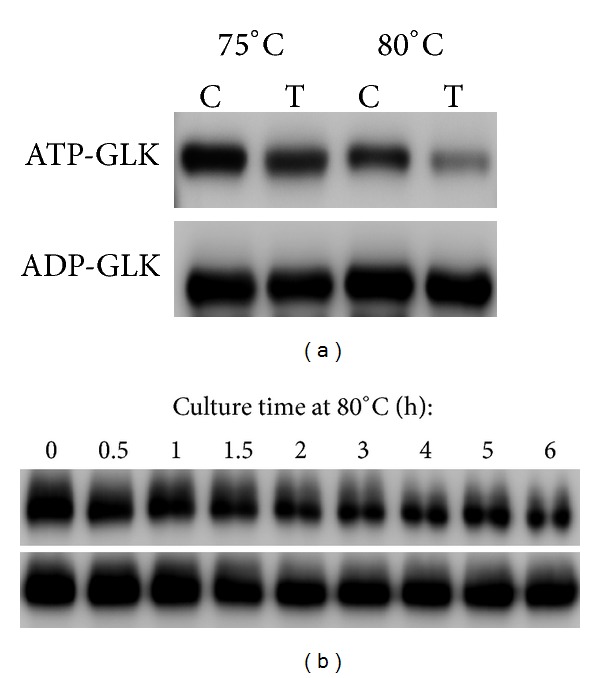
*In vitro *(a) and *in vivo* (b) protein degradation assays of ATP- and ADP-GLK by Western blot. (a) Recombinant ATP- or ADP-GLK was incubated with (T) or without (C) *T*. *tengcongensis* native protein lysate at 75°C or 80°C for 60 min. The reaction was then stopped by addition of SDS loading buffer. After SDS-PAGE separation, the remaining protein was detected by Western blot with specific anti-His6 antibody. (b) *T*. *tengcongensis* cells cultured at 75°C to log phase (set as time 0 h) were transferred to 80°C for further culturing and then collected at the indicated time points to get soluble proteins for Western blot analysis with specific anti-ATP-GLK or anti-ADP-GLK antibodies.

As ATP- and ADP-GLK share the same sugar substrate, glucose, a question arose why *T*. *tengcongensis* possesses two GLKs with similar catalytic functions. One of the apparent answers is that the two enzymes are possibly expressed at different levels under certain circumstances. The abundant status of ATP- and ADP-GLK at three different culture temperatures was evaluated by Western blot, as depicted in Figure 2. The ATP-GLK abundance continuously decreased during a temperature increase from 60 to 80°C, whereas the ADP-GLK abundance was not found to undergo significant changes. This was possibly resulted from two causes, lower expression or higher degradation in response to increased temperature. We took real-time PCR to check the mRNA levels of *TTE0090* and *TTE1961* at different temperatures and found no significant changes in transcriptional level due to the alternated temperature (data not shown). Thus, we came to a hypothesis that the ATP-GLK protein was unstable at higher temperature due to enhanced proteolysis. Protein degradation assays *in vivo* and *in vitro* were carried out to test this hypothesis. After incubation with the *T*. *tengcongensis* lysate at indicated temperatures, the immunoband intensity of the recombinant ATP-GLK appears significantly reduced at 80°C (Figure 3(a) and Supplementary Figure 3). On the other hand, the immunosignals of the recombinant ADP-GLK remain consistent at either 75 or 80°C. The same phenomena were observed in the *in vivo* samples. The *T*. *tengcongensis* harvested from 75°C was immediately reincubated at 80°C with different time followed by Western blot analysis to ATP-GLK and ADP-GLK. The heat shock process indeed caused a change in abundance of the ATP-GLK protein. In Figure 3(b), an obvious attenuation of the immunoband intensity against ATP-GLK emerged after 2 hrs of heat shock treatment, whereas no significant change was found for ADP-GLK. As compared with ADP-GLK, therefore, the ATP-GLK protein seems quite sensitive to temperatures over 75°C, possibly due to activation of proteolysis at high temperatures. 

### 3.3. Involvement of HSP60 in GLK Stability

**Figure 4:**
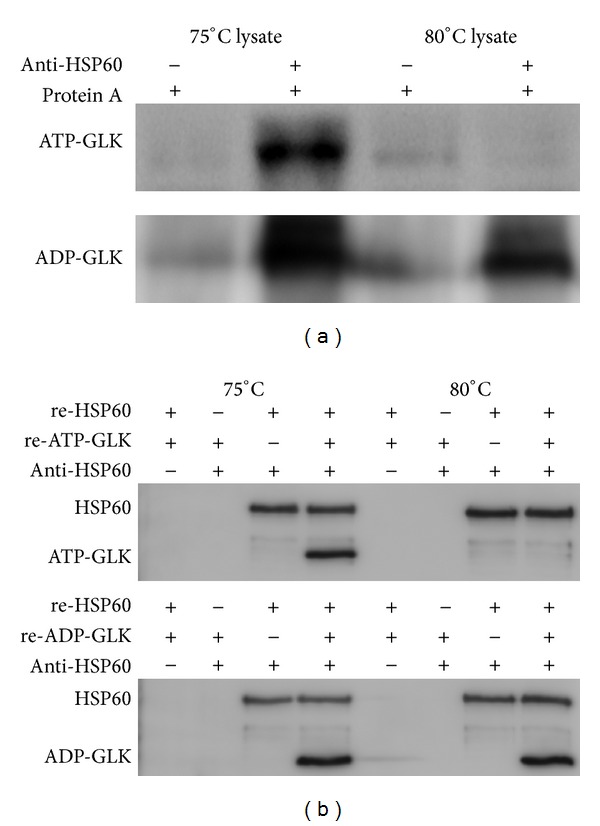
*In vivo* (a) and *in vitro* (b) analysis of interactions between GLKs and HSP60. (a) Co-IP analyses of the interactions between GLKs and HSP60. Anti-HSP60 antibody was used as the bait. After incubation with the native protein lysate either from *T*. *tengcongensis* cells cultured at 75°C or 80°C, the precipitated complexes were identified by Western blot with specific anti-ATP-GLK or anti-ADP-GLK antibody. (b) *In vitro* protein interaction assay of GLKs and HSP60. Recombinant HSP60 was incubated with recombinant ATP-GLK or ADP-GLK in the binding buffer at different temperatures for 30 min. The binding complexes were then precipitated using anti-HSP60 antibody and detected with specific anti-His6 antibody.

HSP is regarded as playing a pivotal role in protein stability, as it protects against protein misfolding and degradation. HSP60 expression in *T*. *tengcongensis* was tightly correlated with temperature, and this protein was able to interact with many proteins and form multiple protein complexes in response to temperature elevation [5]. We asked whether HSP60 could interact with the ATP- or ADP-GLK to protect them from the proteolytic attacks at higher temperatures. Co-immunoprecipitation (Co-IP) experiments using the anti-HSP60 antibody as the bait were employed to isolate the possible complexes of HSP60/ATP-GLK or HSP60/ADP-GLK. As shown in Figure 4(a), the Co-IP products elicited from the lysates of *T*. *tengcongensis* cultured at 75°C contained the two GLKs, whereas only ADP-GLK presented in that at 80°C. This implies that the interactions between HSP60 and ATP-GLK are greatly weakened or that the proteolysis against ATP-GLK is dramatically enhanced at 80°C. In fact, the ATP-GLK abundance is very low at 80°C (Figure 2); thus, it is not easy to evaluate the interactions between HSP60 and ATP-GLK *in vivo*. To specifically clarify the interactions, the recombinant HSP60, ATP-GLK and ADP-GLK were used in the protein interaction assay *in vitro*. The results displayed in Figure 4(b) reveal that the recombinant ATP-GLK and ADP-GLK share similar affinities to HSP60 at 75°C. Nevertheless, there was no interaction between ATP-GLK and HSP60 at 80°C, even though the ADP-GLK/HSP60 interaction remained unchanged. In the examination systems *in vitro,* there was no native component of *T*. *tengcongensis* participating in the protein interactions; thus, we hypothesize that the weakened interactions between HSP60 and ATP-GLK at 80°C lead to instability of the ATP-GLK protein at high temperature.

## 4. Discussion

All the GLKs discovered are grouped into three evolutionarily disconnected clusters, including the mammalian GLKs, the yeast GLKs, and the bacterial GLKs [24, 26]. The bacterial GLK family is comprised of enzymes of about 40 kDa and found exclusively in Eubacteria. This family could be subdivided into three groups, proteobacteria together with cyanobacterium (Group A), gram-positive bacteria (Group B), and fructokinases (Group C) [27]. According to the phylogenetic analysis of GLKs from different sources (see Supplementary Figure 4), ATP-GLK in *T*. *tengcongensis* is grouped into Group B, together with several thermophilic glucokinases, while the mesophilic ATP-GLKs are generally grouped into Group A, as reported by Wu et al. [27]. In 1995, a novel GLK with ADP as the phosphoryl donor was characterized in *Pyrococcus furiosus* [24], the first report of ADP-GLK. The crystal structure of ADP-GLK from *Thermococcus litoralis* bound with ADP revealed that the overall structure could be divided into large and small alpha/beta domains, and the ADP molecule was buried in a shallow pocket in the large domain [18]. The phylogenetic analysis to ADP-GLK is still restricted because of few such enzymes found so far. For instance, the ADP-GLKs in *Bos Taurus* and *Dictyostelium discoideum* are similar to the archaeon ADP-GLKs; however, the *T*. *tengcongensis* ADP-GLK seems distinct from the archaeon ADP-GLKs. 

A fundament question arose in this study is why *T. tencongensis* needs the two different GLKs. It is known that the optimal temperature for* T*. *tengcongensis *is 75°C, while 80°C is close to the upper limit. All the evidence in our study revealed that ATP-GLK in this thermophile only performs 100% of enzyme activity at 75°C but decreased dramatically at 80°C, while ADP-GLK only exhibits 100% of activity at 80°C. And, in contrast to the substrate specificity and ionic tolerance of ADP-GLK, ATP-GLK displays relatively wide selections (Table 1). The different performance of the two GLKs in *T*. *tengcongensis* leads to a conclusion that ATP-GLK is a better choice for this bacterium grown at 75°C, and ADP-GLK is a better choice at 80°C. The conclusion seems quite reasonable. When a life species is living with harsh conditions, conservation of energy consumption is always challengeable. We found that ATP generation was slow down in *T. tencongensis *at the temperature as high as 80°C, according to the energy charge detected (data not shown). Moreover, the ATP hydrolysis rate is supposed much fast at such high temperatures [20, 24]. If *T. tencongensis* could utilize ADP-GLK in glycolysis pathway at 80°C, its ATP consumption is expected to be reduced significantly. This will be helpful for the bacteria having enough ATP to survive at higher temperature (80°C). In another words, this is the result of nature selection. 

Interestingly, our data suggested that the protein abundance of ATP-GLK decreased at 80°C due to the protein proteolysis, while the abundance of ADP-GLK keep stable as the temperature elevated 80°C. As we know, a cell will suffer stress when it confronts with an abrupt change in its immediate surroundings. Protein damage and gene down-regulation are only parts of the stress response. There is yet another important component of the stress response-activation of the stress genes. Stress proteins are reported to be multifunctional and ubiquitous. They play important roles in all cells, cell compartments, and organelles and are said to be promiscuous as they interact with a great variety of other molecules [28]. HSP60, or GroEL, is widely considered to be a chaperone protein that protects proteins from degradation, denaturation, or misfolding [29, 30]. The investigation of the HSP60 chaperone complexes of *E*. *coli* revealed that HSP60 could promote the proper folding of UmuC protein *in vitro* and help to recover its DNA binding activity [31]. Stan et al. [32] developed a sequence-based approach to identify the natural substrate proteins (SPs) for HSP60. Over 50% of proteins were the putative SPs of GroEL in *E*. *coli*, *Saccharomyces cerevisiae*, *Thermoplasma acidophilum*, *Methanopyrus kandleri* and *Ureaplasma urealyticum*. In *Thermoanaerobacter brockii*, Truscott et al. [33] observed that HSP60 and HSP10 together were active in protein-folding assays, and three enzymes, malate dehydrogenase, isocitrate dehydrogenase, and alcohol dehydrogenase, were protected from aggregation by association with the two chaperonins at 60 to 65°C. Meng et al. [5] performed the proteomic analysis of temperature-dependent complexes in *T*. *tengcongensis*. Using blue native polyacrylamide gel electrophoresis (BN-PAGE) combined with MALDI TOF/TOF MS or LC MS/MS, HSP60 was globally identified in all six temperature-dependent complexes. The most abundant proteins identified in all six complexes were carbohydrate metabolism-related enzymes, about 30% of all identified proteins. And we found that ATP-GLK and ADP-GLK are new substitutes of HSP60, who is responsible for their stability under different temperatures. At 80°C, HSP60 could not bind with ATP-GLK, which results in the unprotection of ATP-GLK. Then, the ATP-GLK is sensitive to high temperature (80°C) and easy to be degraded. Therefore, the ADP-GLK will be the only glucokinase to use for *T*. *tengcongensis* growing at 80°C, which can be helpful to efficient survival. This may also indicate a brand new area in which HSP60 is involved.

## 5. Conclusions

In this communication, we primarily characterized the three GLKs in *T. tengcongensis. *We furthermore focus on the question why *T. tengcongensis* possess two GLKs, ATP- and ADP-GLK. Above the optimal temperatures, ATP-GLK was found reduction of its catalytic activity and thermal stability, whereas ADP-GLK was confirmed relatively stable. Moreover, degradation rates of ATP-GLK and ADP-GLK in *T. tengcongensis* were evaluated, indicating that ATP-GLK was unstable due to weaken interactions with HSP60 at 80°C. We thus conclude that* T. tengcongensis *prefers ADP-GLK in glycolysis at higher temperature. 

## Supplementary Material

Supplementary Figure 1. Multi-sequence alignment of glucokinase candidates from T. tengcongensis compared to GLK from T. maritima. The boxed regions are possible conserved motifs.
Supplementary Figure 2. Temperature-dependence of recombinant ATP- and ADP-GLK. The enzyme-specific activities of recombinant ATP- and ADP-GLK were tested at different temperatures, from 30 to 95 °C. One hundred percent of activity corresponded to the specific activity at 75 °C (ATP-GLK) or 80 °C (ADP-GLK).
Supplementary Figure 3. Dynamic analysis of in vitro protein degradation of recombinant GLKs. Upper panel: Recombinant ATP- or ADP-GLK was incubated with (T) or without (C) T. tengcongensis native protein lysate at 80 °C for the indicated times. Then, the reactions were stopped by addition of SDS loading buffer. After SDS-PAGE separation, the remaining protein was detected by Western blot with specific anti-His6 antibody. Lower panel: the abundance of each immune band was estimated by image quantification software. The ratios between T and C were calculated and plotted, as shown.
Supplementary Figure 4. Phylogenetic analysis of glucokinases from different sources, using the maximum likelihood method. The horizontal bar represents a distance of 0.2 substitutions per site. The values above the lines are bootstrap values. Only values higher than 40 are shown. Database accession numbers or local gene tags are shown after the species names.

## Figures and Tables

**Table 1:** Biochemical and kinetic properties of kinases of ROKs in *T*. *tengcongensis. *

Parameter	pH optimum	Apparent *T*_opt_ (°C)	Apparent *K*_*m*_ (mM)		Apparent *V*_max⁡_ (U/mg)		Sugar specificity (*V*_max⁡_%)				Cation specificity (*V*_max⁡_%)		Phosphoryl donor specificity (*V*_max⁡_%)	
			Glucose/gluNac	ATP/ADP	Glucose/gluNac	ATP/ADP	2-deoxyglucose	Fructose, galactose	Glucose, mannose	galNac, gluNac, manNac	Ca^2+^, Cu^2+^, Mg^2+^	Ni^2+^, Sr^2+^, Zn^2+^	ADP, ATP, CTP	GTP, ITP, PEP
*TTE0090*	8.0	75	2.4/ND	2.6/ND	90/ND	100/ND	13.5	3, 0	100, 99	0, 0, 0	13.2, 0.4, 100	69.1, 17, 34	3, 100, 38	96, 87, 0
*TTE1961*	8.0	80	2.1/ND	ND/0.9	93/ND	ND/106	1.2	0, 0	100, 17	0, 0, 0	ND, ND, 100	ND, ND, ND	100, 0, ND	ND, ND, ND
*TTE2418*	7.5	70	ND/0.9	1.9/ND	ND/229	51/ND	0	0, 6.5	23.9, 6.5	0, 100, 99.8	2.6, 0, 100	5.2, 3.5, 11.3	7, 100, 7	15.5, 57.7, 0
