# Supplementary material for: Thermal Stability of Glucokinases in Thermoanaerobacter tengcongensis
Source: Biomed Res Int. 2013 Aug 24;2013:646539. doi: 10.1155/2013/646539 (PMC3766608; doi:10.1155/2013/646539)

Figure legends

Supplementary Figure 1. Multi-sequence alignment of glucokinase candidates from *T. tengcongensis* compared to GLK from *T. maritima*. The boxed regions are possible conserved motifs.

Supplementary Figure 2. Temperature-dependence of recombinant ATP- and ADP-GLK. The enzyme-specific activities of recombinant ATP- and ADP-GLK were tested at different temperatures, from 30 to 95 ℃. One hundred percent of activity corresponded to the specific activity at 75 ℃ (ATP-GLK) or 80 ℃ (ADP-GLK).

Supplementary Figure 3. Dynamic analysis of *in vitro* protein degradation of recombinant GLKs. Upper panel: Recombinant ATP- or ADP-GLK was incubated with (T) or without (C) *T. tengcongensis* native protein lysate at 80 ℃ for the indicated times. Then, the reactions were stopped by addition of SDS loading buffer. After SDS-PAGE separation, the remaining protein was detected by Western blot with specific anti-His6 antibody. Lower panel: the abundance of each immune band was estimated by image quantification software. The ratios between T and C were calculated and plotted, as shown.

Supplementary Figure 4. Phylogenetic analysis of glucokinases from different sources, using the maximum likelihood method. The horizontal bar represents a distance of 0.2 substitutions per site. The values above the lines are bootstrap values. Only values higher than 40 are shown. Database accession numbers or local gene tags are shown after the species names.

Supplementary Figure 1

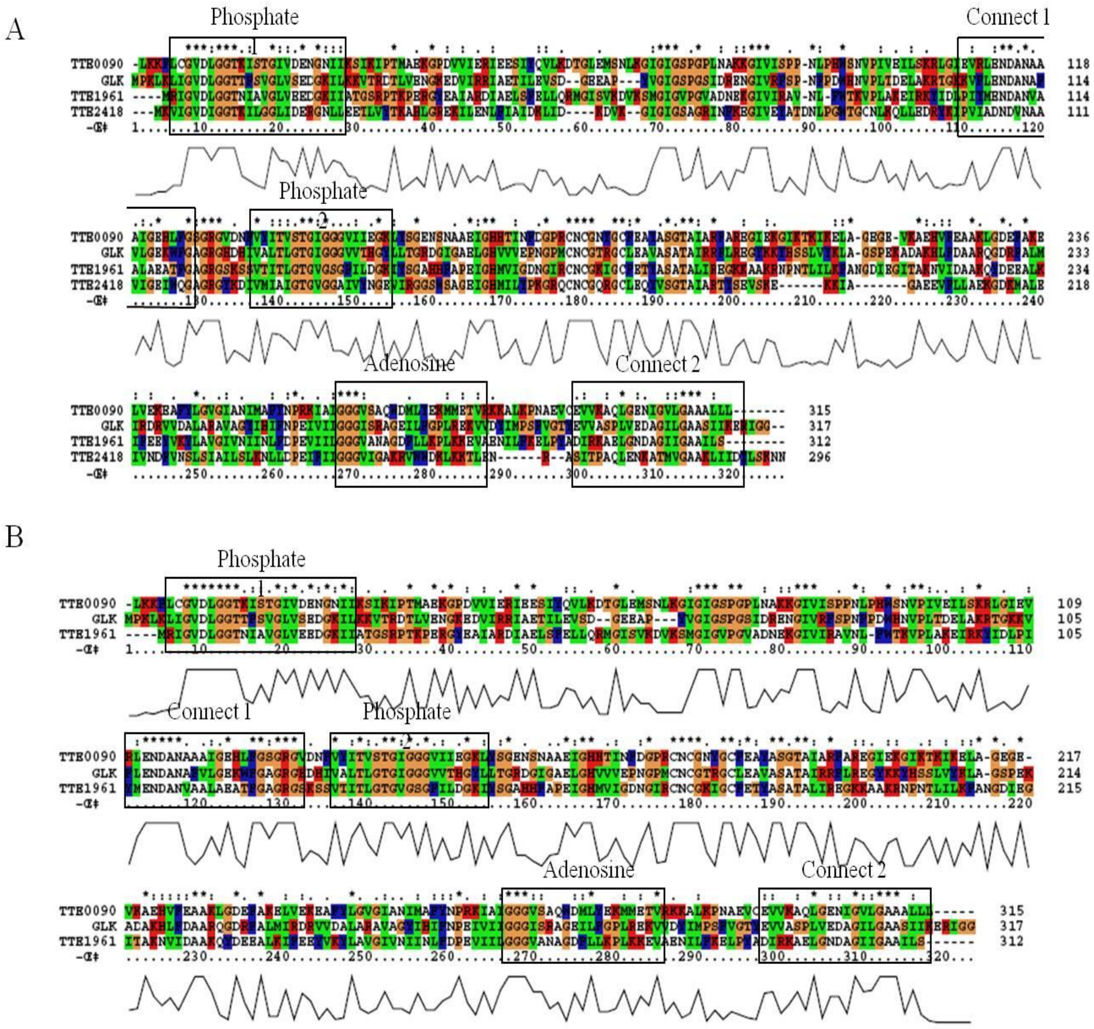

Supplementary Figure 2

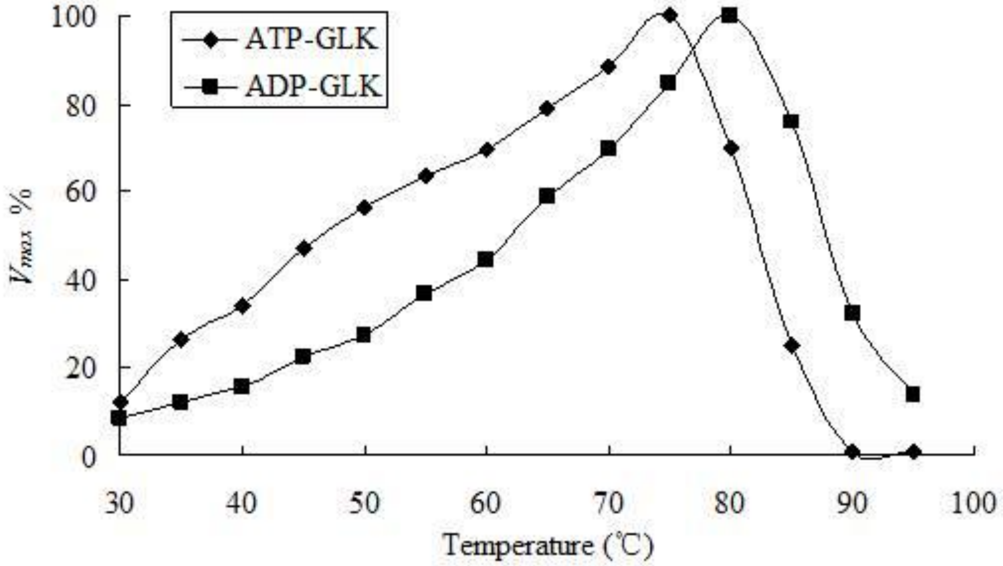

Supplementary Figure 3

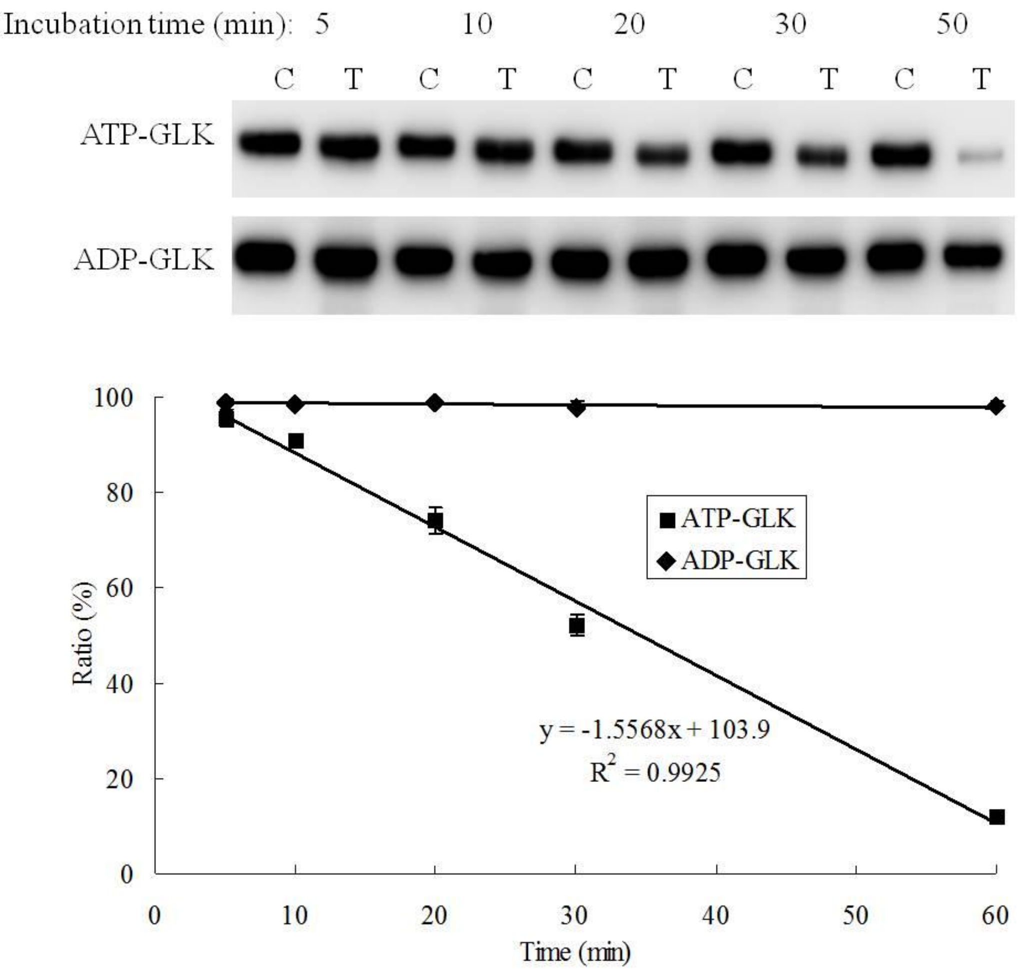

Supplementary Figure 4

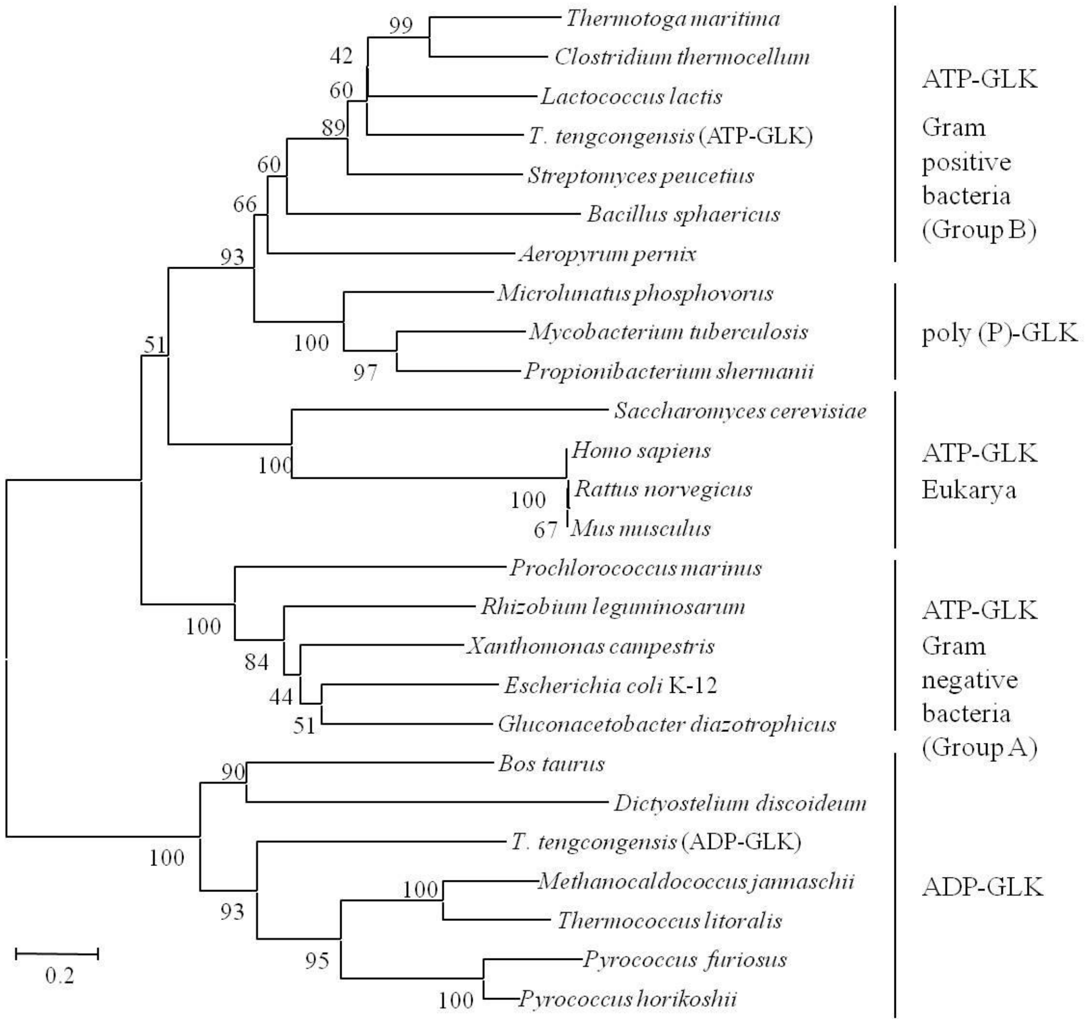

Supplement: Supplementary file 1 — Supplementary Figure 1. Multi-sequence alignment of glucokinase candidates from T. tengcongensis compared to GLK from T. maritima. The boxed regions are possible conserved motifs. Supplementary Figure 2. Temperature-dependence of recombinant ATP- and ADP-GLK. The enzyme-specific activities of recombinant ATP- and ADP-GLK were tested at different temperatures, from 30 to 95 °C. One hundred percent of activity corresponded to the specific activity at 75 °C (ATP-GLK) or 80 °C (ADP-GLK). Supplementary Figure 3. Dynamic analysis of in vitro protein degradation of recombinant GLKs. Upper panel: Recombinant ATP- or ADP-GLK was incubated with (T) or without (C) T. tengcongensis native protein lysate at 80 °C for the indicated times. Then, the reactions were stopped by addition of SDS loading buffer. After SDS-PAGE separation, the remaining protein was detected by Western blot with specific anti-His6 antibody. Lower panel: the abundance of each immune band was estimated by image quantification software. The ratios between T and C were calculated and plotted, as shown. Supplementary Figure 4. Phylogenetic analysis of glucokinases from different sources, using the maximum likelihood method. The horizontal bar represents a distance of 0.2 substitutions per site. The values above the lines are bootstrap values. Only values higher than 40 are shown. Database accession numbers or local gene tags are shown after the species names. [file 646539.f1.pdf]
